# Short-term monocular pattern deprivation reduces the internal additive noise of the visual system

**DOI:** 10.3389/fnins.2023.1155034

**Published:** 2023-07-27

**Authors:** Jinwei Li, Zhenhui Cheng, Jing Li, Linghe Li, Lijun Chen, Jiayu Tao, Zeng Wang, Di Wu, Pan Zhang

**Affiliations:** ^1^Department of Psychology, Hebei Normal University, Shijiazhuang, China; ^2^Department of Psychology, Shandong Normal University, Jinan, China; ^3^Department of Psychology, Chengde Medical University, Chengde, China; ^4^Department of Psychology, Hebei Medical University, Shijiazhuang, China; ^5^Military Medical Psychology School, Air Force Medical University, Xi’an, China

**Keywords:** monocular pattern deprivation, contrast sensitivity, external noise, perceptual template model, spatial frequency, internal additive noise

## Abstract

Previous studies have shown that short-term monocular pattern deprivation can shift perceptual dominance in favor of the deprived eye. However, little is known about the effect of monocular pattern deprivation on contrast sensitivity (CS) and its corresponding mechanisms. Here, contrast sensitivity function (CSF) in the nondominant eye of normal subjects was evaluated before and after 150 min of monocular pattern deprivation. To obtain a CSF with high precision and efficiency before deprivation effect washout, a quick CSF (qCSF) method was used to assess CS over a wide range of spatial frequencies and at two external noise levels. We found that (1) monocular pattern deprivation effectively improved the CS of the deprived eye with larger effect on high spatial frequencies, (2) CS improvement only occurred when external noise was absent and its amount was spatial frequency dependent, and (3) a perceptual template model (PTM) revealed that decreased internal additive noise accounted for the mechanism of the monocular pattern derivation effect. These findings help us better understand the features of short-term monocular pattern deprivation and shed light on the treatment of amblyopia.

## Introduction

Neuroplasticity, which refers to the ability to modify neural circuits to adapt to environmental changes under the influence of experience, is a fundamental property of the nervous system (Wiesel, 1983; Pascual-Leone et al., 2005). Before critical period (7–9 years), visual deprivation resulted in impaired visual acuity (Freeman and Bradley, 1980), spatio-temporal sensitivity (Hess et al., 1981), as well as shape and depth perception (Fine et al., 2002, 2003) in individuals with untreated congenital cataracts. Patching therapy is based on the principle of visual deprivation. Researchers force amblyopia to use lazy eye by covering their fellow eye (Ramamurthy and Blaser, 2021).

It is widely accepted that the brain undergoes limited plasticity beyond the critical period, with minimal experience-dependent changes. For instance, patching therapy was traditionally considered effective for amblyopia before 7–9 years of age. However, recent studies have questioned this notion. Using the classic binocular rivalry paradigm (in which different visual stimuli are presented to both eyes at the same time), researchers have found that 150 min of monocular deprivation, which eliminated pattern information but retained mean luminance (for short, we use the term “monocular pattern deprivation, MPD” in this article), shifted perceptual dominance to the deprived eye (Lunghi et al., 2011). This effect can be validated in a variety of binocular tasks, including the binocular phase combination task, dichoptic global motion coherence task, and binocular contrast matching task (Zhou et al., 2013a). The same was true when researchers used other methods of deprivation, such as opaque patching (eliminating both pattern information and mean luminance), continuous flash suppression (Kim et al., 2017), and kaleidoscopic deprivation (Ramamurthy and Blaser, 2018). Moreover, studies have shown that the MPD effect in the normal group is smaller than that in the adult amblyopic group, which suggests that the amblyopic visual cortex has a greater degree of plasticity (Zhou et al., 2013b). In addition, researchers investigated the cumulative effects of MPD on adults and old children with amblyopia and observed a long-term improvement in visual functions (Zhou et al., 2019). The researchers even recommended MPD as a conventional treatment for amblyopia. Given that MPD is not only low-cost but also convenient to operate, here, we paid attention to the effect of MPD on visual function.

Technically speaking, monocular deprivation can be considered a form of low-contrast adaptation or contrast deprivation for one eye. The impact of monocular deprivation can be viewed as a unique type of contrast adaptation, where the average contrast level diminishes close to zero (in the case of a translucent patch) or reaches zero (in the case of an opaque patch) in the affected eye. However, one recent review demonstrated that contrast adaptation is fundamentally a between-eye effect, whereas monocular deprivation is fundamentally a within-eye effect (Hess and Hyun Min, 2023). Their underlying neural mechanisms are expected to differ. For examples, following low-contrast adaptation, both eyes benefit, although the adapted eye experiences a greater advantage (Kwon et al., 2009). In contrast, monocular deprivation enhances the effectiveness of the deprived eye, while diminishing the effectiveness of the other eye (Zhou et al., 2013a). The changes after contrast deprivation is orientationally tuned (Zhang et al., 2009), while monocular deprivation is not (Zhou et al., 2014; Wang et al., 2017). Furthermore, the effect of the duration of adaptation seems to be uniquely different between contrast adaptation and monocular deprivation (Min et al., 2022).

The measure of CS has fundamental implication for visual sciences, as it can be used to distinguish between visible and invisible objects, i.e., defines the threshold (Pelli and Bex, 2013). Many studies concerning the retina, glaucoma, and neuro-ophthalmology have shown that CS deficits were evident even when individuals’ performance on visual acuity tests appeared normal (Jindra and Zemon, 1989; Woods and Wood, 1995; Pelli and Bex, 2013). Thus, there is no denying that the CS measure is a better predictor of functional vision than visual acuity in clinical applications. In reviewing previous studies, the measurement of CS was rarely used for evaluating the MPD effects. Specifically, Zhou et al. measured the effect of MPD on the monocular contrast threshold (Zhou et al., 2013b). Nevertheless, the MPD effect was investigated at only one spatial frequency (i.e., 0.3 cpd), and only two subjects were involved, so the results lacked generalization. The contrast sensitivity function (CSF) indicates sensitivity (1/threshold) as a function of spatial frequency, and it allows a comprehensive characterization of human vision. Therefore, it is necessary to systematically investigate the effect of MPD on CS over a broad range of spatial frequencies. However, a long testing duration is required to measure the whole CSF by traditional methods, e.g., 3 down/1 up staircases, and the peak of short-term MPD effects lasts only approximately 10 min (Zhou et al., 2013a, 2014; Lunghi et al., 2015). Using the constant stimuli method, Zhou et al. (2017) investigated the effect of MPD on the contrast threshold for achromatic and chromatic stimuli, with each measurement requiring 15 min and conducted at a single spatial frequency (Zhou et al., 2017). Recently, Chen et al. (2023) reported that internal neural states can affect the short-term impact of MPD on contrast threshold at 2 cycles per degree (cpd) (Chen et al., 2023). However, it should be noted that the adjustment method in this study may lead to an overestimation of the threshold by the subjects. Fortunately, Lesmes et al. proposed the quick CSF (qCSF) algorithm for accurately and precisely estimating spatial CSF (Lesmes et al., 2010). This algorithm immensely improves the measurement efficiency by combining Bayesian adaptive inference with a trial-to-trial information gain strategy, allowing us to measure the effect of MPD on the CSF during its peak period. Thus, the first aim of the current study was to investigate the effect of MPD on monocular CSF by taking advantage of the qCSF algorithm.

The perceptual template model (PTM) is an ideal tool for characterizing the psychophysical mechanism of visual function alteration. In the context of the PTM, there are two types of internal noise that can affect sensory processing: internal additive noise and internal multiplicative noise. Internal additive noise refers to a source of variability that adds an independent random component to the neural activity representing sensory stimuli. In contrast, internal multiplicative noise refers to a source of variability that scales the neural activity representing sensory stimuli. Rather than adding a constant value to the response, this noise multiplies the response by a scaling factor that varies randomly over time. The key difference between internal additive noise and internal multiplicative noise lies in how they affect information processing in the brain. Additive noise has the effect of reducing the signal-to-noise ratio of sensory responses, making it more difficult to distinguish between different stimuli. Multiplicative noise, on the other hand, changes the shape of the response distribution, making it more skewed and elongated. This can have important effects on the perceived similarity of stimuli, the tuning of individual neurons, and the coding efficiency of neural populations (Lu and Dosher, 1999; Pelli et al., 2006; Rhodes and Vargas, 2014). In the context of PTM, external noise images are added to the signal, requiring observers to eliminate the interference caused by these external noise images. Improved observers’ performance through MPD may lead to the reduction of internal additive noise, optimization of perceptual template for improved external noise exclusion, or a decrease in internal multiplicative noise (in accordance with Weber’s law) (Dosher and Lu, 1998).

By varying different external noise levels (mask images) and measuring the corresponding CSF, researchers have explored the mechanisms of system changes after perceptual learning (Dosher and Lu, 1998; Bower and Andersen, 2012; Zhang et al., 2018), long-term action video games playing (Bejjanki et al., 2014), and attention modulation (Lu and Dosher, 1998; Dosher and Lu, 2000). Thus, the second aim of the current study was to quantify the psychophysical mechanisms underlying MPD-induced CS improvement using the external noise method and PTM approach.

To achieve the above objectives, the qCSF procedure was adopted in the current study to evaluate the short-term MPD effects on monocular visual function. Specifically, the following two assessments were performed. First, we measured CSF twice with a grating detection task before and immediately after MPD to verify whether the CS of the deprived eye increased and to determine how CS improvement was modulated by spatial frequency and the external noise level. Second, we examined the psychophysical mechanisms underlying MPD-induced CS enhancement.

## Methods

### Subjects

*A priori* power analysis was conducted with G*Power 3.1.9.7 to determine the required sample size (Faul et al., 2007). We set the effect size (*η*^2^) of the deprivation effect as 0.251 according to a previous study (Ramamurthy and Blaser, 2021). It showed that at least seven subjects were needed to obtain a power of 0.8. Therefore, we recruited nine undergraduate students aged 19–23 with normal or well-corrected (≥20/20) vision. Prior to participating in the experiment, each subject underwent the hole-in-the-hand test to determine which eye was the dominant eye. All subjects were not aware of this study’s purpose and expressed their informed consent before participating in the experiment. The Ethical Review Committee of Hebei Normal University authorized all procedures, which were carried out in compliance with the Declaration of Helsinki.

### Apparatus

The stimuli were displayed on a luminance-calibrated cathode-ray tube (CRT) monitor (brand Dell; resolution 1,280 × 1,024; fresh rate 85 Hz). The Psychophysics Toolbox in MATLAB was used to operate the monitor (Pelli, 1997). The subjects rested their heads on a chin rest and sat comfortably at a distance of 171 cm from the screen. The background brightness of the stimuli was set to 41.6 cd/m^2^. Except for the test phase, the subjects wore an apparatus made by modifying goggles. To produce the MPD effect, a translucent plastic material was attached to the side of the goggles. It allowed light to reach the retina (attenuation 15%), but no pattern information (Lunghi et al., 2011). The middle of the goggles was sealed with clay to isolate any residual pattern information.

### Stimuli

In the qCSF test, vertical gratings were exhibited in the central visual field as targets. The spatial frequencies of gratings varied at ten levels (0.5, 0.67, 1, 1.33, 2, 2.67, 4, 5.33, 8, and 16 cpd). Noise images were used for interference target detection and generated from a uniform normal distribution [*μ* = 0 and *σ*∈ = 0.24]. The cycle number of gratings was constant (*n* = 3); thus, there was a high spatial frequency along with a small size. The noise image is a type of “external noise” in the context of visual perception or psychophysics (Dosher and Lu, 1998; Lu and Dosher, 1998; Dosher and Lu, 2000; Xu et al., 2006; Bower and Andersen, 2012; Zhang et al., 2018). The sizes of the gratings and noise images were identical, varying between ten sizes (576, 432, 288, 216, 144, 108, 72, 54, 36, and 18 pixels). In each trial, the size of noise image was identical to the grating and contained 15 × 15 noise elements (Zhang et al., 2018, 2021). To blur the edge of gratings, the gratings were covered by truncated Gaussian envelopes.

### Procedure

**Figure 1:**
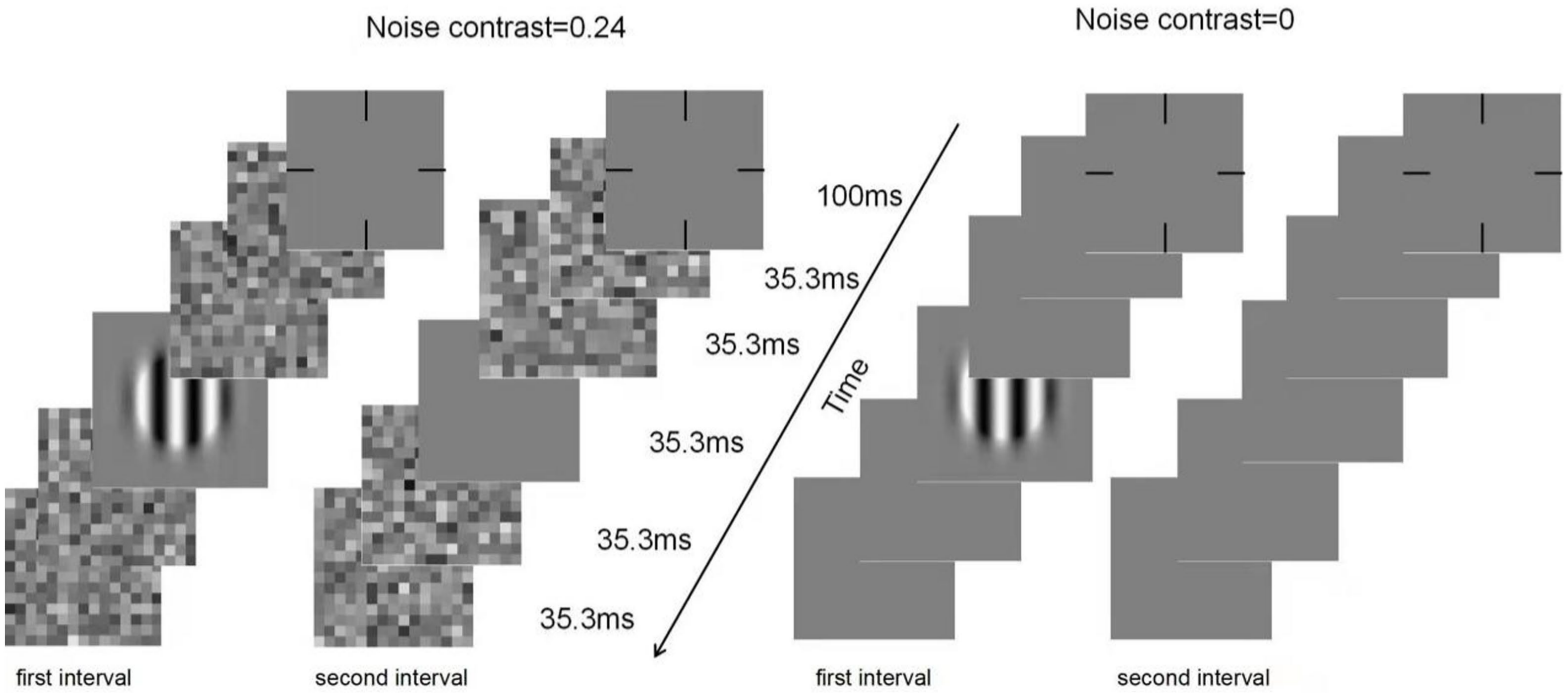
Illustration of a typical trial in the qCSF procedure under zero- (right) and high-(left) noise conditions.

CSF was assessed by a contrast detection task. There were two noise levels (0 and 0.24) and ten spatial frequency conditions. The ‘noise level × spatial frequency’ condition was randomly mixed between trials. Each noise level contained 75 trials. Each trial was divided into two intervals by a 500 ms blank screen. With a ‘ding’ sound, each interval began with a 100 ms cross, followed by five frames of images, each lasting 35.5 ms. In the zero-noise condition, a grating was present in the middle frame of one interval. Using a gaming controller, subjects were instructed to point out which interval contained the grating. Each response was followed by a brief beep. In the high-noise condition, the first and last two blanks in each interval were replaced by noise images that were randomly generated from the same noise distribution. Figure 1 shows an illustration of a typical trial.

### Experimental design

**Figure 2:**
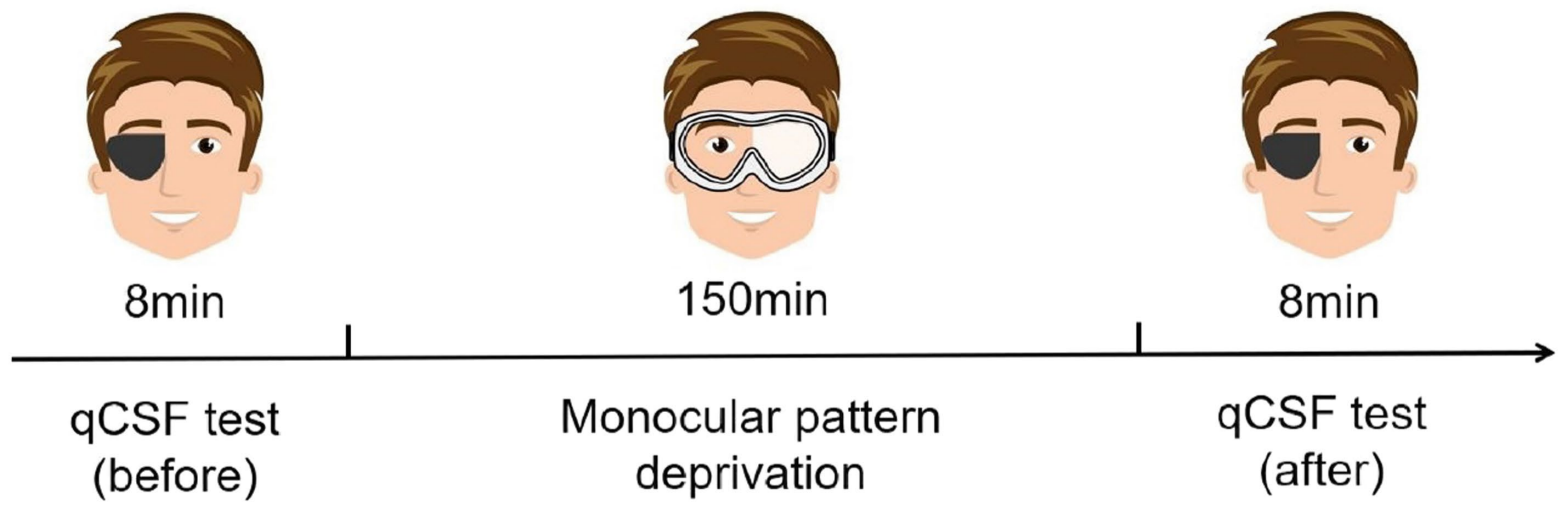
Schematic diagram of the experimental procedure (for subjects with the right eye as the dominant eye).

It was found that MPD could be a potential method to enhance the vision of the lazy eye (non-dominant eye) among patients with myopia (Zhou et al., 2019). From an application perspective, although depriving the dominant eye may result in a stronger effect (Lunghi et al., 2011), we intervened on the nondominant eye for a more balanced binocular function. The experiment contained three stages (Figure 2). In the first stage, subjects needed to complete the qCSF test with their nondominant eyes. This stage took approximately 8 min. In the second stage, subjects’ nondominant eyes were covered with translucent plastic material for 150 min. During MPD, subjects were allowed to engage in daily activities around the laboratory. In the third stage, subjects needed to complete the qCSF test again. Before the formal experiment, subjects practiced hundreds of trials on the qCSF test to ensure that they could complete the task quickly and well.

### Data analysis

All variables were computed in log units. The area under the log contrast sensitivity function (AULCSF) is a summary measure that quantifies the overall sensitivity across a range of spatial frequencies and higher values indicate greater overall sensitivity. We used the trapezoidal rule, which involves breaking the log CSF curve into trapezoids and calculating their areas (Koop et al., 1996; Zhang et al., 2022; Guo et al., 2023). It is worth noting that spatial frequency was computed in log2 units.

The PTM model regards the observer as a system and describes the link between the perceptual input and output and the decision-making process. The model defines subjects’ performance as four parts: nonlinear transfer, perceptual template gain, internal additive noise and internal multiplicative noise (Dosher and Lu, 1998, 1999; Lu and Dosher, 1998). Subjects’ performance was evaluated by Eq. 1 in the PTM:


(1) $$d^{\prime }=\frac{{\left(\beta c\right)}^{\gamma }}{\sqrt{{\left(N_{ext}\right)}^{2\gamma }+N_{mul}^{2}\left({\left(\beta c\right)}^{2r}+{\left(N_{ext}\right)}^{2\gamma }\right)+{\left(N_{add}\right)}^{2}}},$$

where *d*′ is defined as subject performance; *N*_ext_ expresses the external noise contrast; *N*_mul_ and *N*_add_ represent internal multiplicative and additive noise, respectively; *γ* presents the system’s nonlinearity; *β* denotes the gain of the perceptual template; and *c* signifies the contrast threshold. We placed *A*_f_, *A*_m_, and *A*_a_ before *N*_ext_, *N*_mul_, and *N*_add_, respectively, to simulate the impact of MPD on CS (see Eq. 2). *A*_f_ (0 < *A*_f≤_ 1 in linear or *A*_f≤_0 in log units) signifies the enhancement of external noise exclusion capability, *A*_m_ (0 < *A*_m≤_ 1 in linear or *A*_m≤_0 in log units) signifies the attenuation of multiplicative noise, and *A*_a_ (0 < *A*_a≤_ 1 in linear or *A*_a≤_0 in log units) signifies the amplification of both signal and noise.


(2) $$d^{\prime }=\frac{{\left(\beta c\right)}^{\gamma }}{\sqrt{{\left(A_{f}N_{ext}\right)}^{2\gamma }+A_{m}^{2}N_{mul}^{2}\left({\left(\beta c\right)}^{2r}+{\left(A_{f}N_{ext}\right)}^{2\gamma }\right)+{\left(A_{a}N_{add}\right)}^{2}}}$$

We can solve Eq. 2 with a given performance level *d*′ to represent contrast threshold *c_τ_* as a function of *N*_ext_ in log form:


(3) $$\log\left(c_{\tau }\right)=\frac{1}{2\gamma }\left\{\begin{array}{l} \log\left[\left(1+A_{m}^{2}N_{mul}^{2}\right)A_{f}^{2\gamma }N_{ext}^{2\gamma }+A_{a}^{2}N_{add}^{2}\right]\\ -\log\left[\frac{1}{d\prime^{2}}-A_{m}^{2}N_{mul}^{2}\right] \end{array}\right\}-\log\left(\beta \right)\;$$

where *c_τ_* indicates the predicted contrast threshold.

Based on the literature, if the slope of psychometric functions was unchanged after a treatment (e.g., perceptual learning, Xu et al., 2006; Zhang et al., 2018), the multiplicative noise was constant. In this case, *A*_m_ is removed from Eq. 3. Thus, a slope check should be performed before fitting the PTM to the data. A previous study found that *N*_add_ and *β* varied with spatial frequency, but *N*_mul_ and *γ* did not (Chen et al., 2014).

To choose the best model, we computed the goodness of fit (*r*^2^, see Eq. 4) for each nested model:


(4) $$r^{2}=1-\frac{\sum {\left(y_{i}-{\hat{y}}_{i}\right)}^{2}}{\sum {\left(y_{i}-\bar{y}\right)}^{2}},$$

where $y_{i}$ and $\hat{y_{i}}$ present the original and predicted values (CS in log10 unit), respectively, and the average of $y_{i}$ is denoted by $\bar{y}$ .The best-fitting model refers to the model whose *r*^2^ was statistically superior to those of any reduced model but not significantly inferior to the full model (Huang et al., 2010; Zhang et al., 2018):


(5) $$F\left(df_{1},df_{2}\right)=\frac{\left(r_{full}^{2}-r_{reduced}^{2}\right)/df_{1}}{\left(1-r_{full}^{2}\right)/df_{2}},$$

where *df*_1_ = *k*_full_ − *k*_reduced_, *df*_2_ *= N* − *k*_full_, *N, k*_full,_ *k*_reduced_ are the number of data points, the numbers of parameters of the full and reduced models, respectively.

## Results

**Figure 3:**
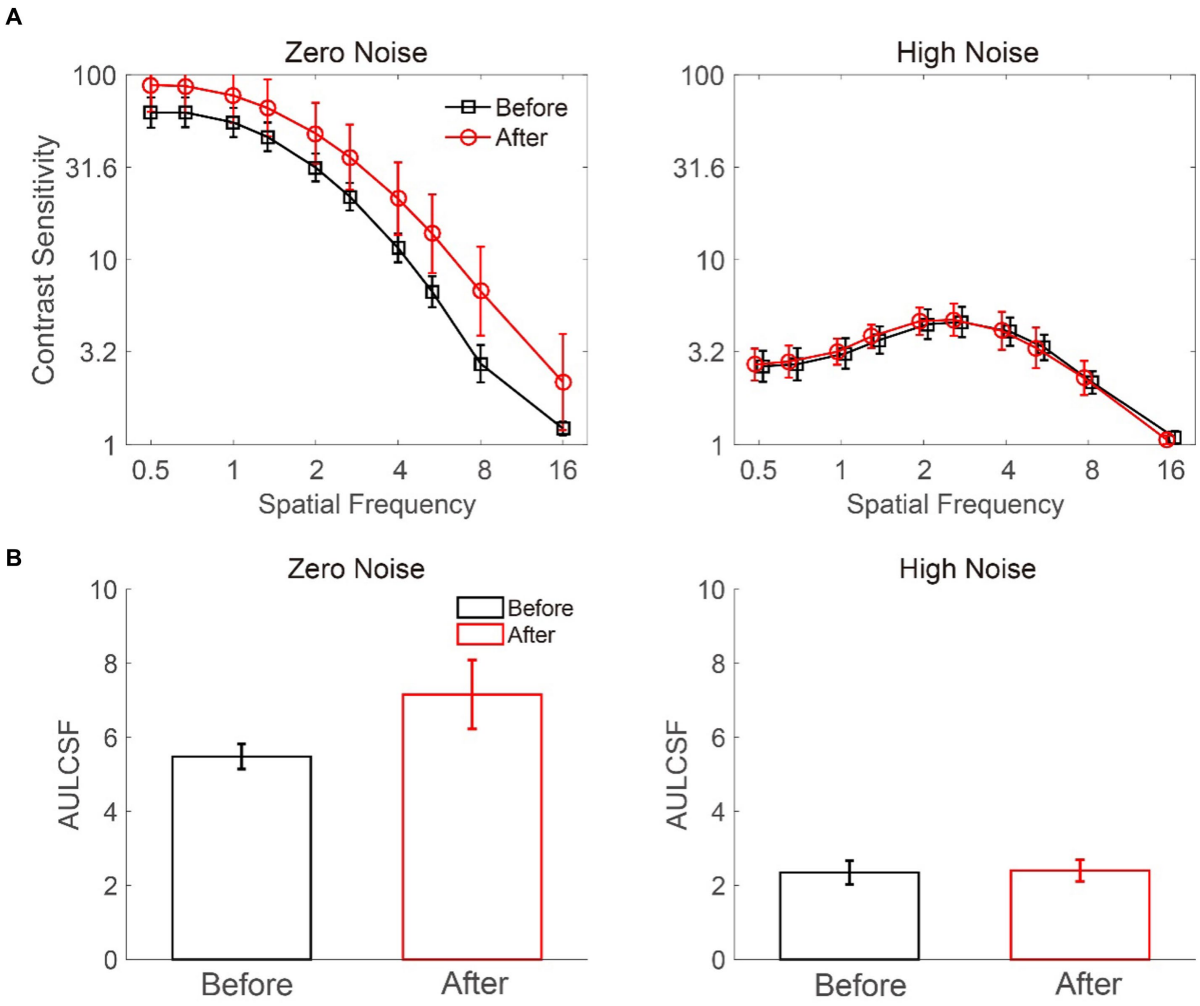
Average CSF **(A)** and AULCSF **(B)** at zero and high noise levels. The black and red colors denote the data before and after MPD, respectively. The error bars represent the standard error.

We measured the CSF of deprived eyes before and after MPD for each participant (Figure 3A). A 2 × 2 × 10 repeated-measures ANOVA was conducted to evaluate log CS with the external noise level (zero vs. high), deprivation stage (before vs. after), and log spatial frequency (from 0.5 to 16 cpd) as independent variables. The results showed a significant main effect of noise level (*F* (1, 8) = 50.793, *p* < 0.001) and spatial frequency (*F* (9, 72) = 94.486, *p* < 0.001) but only a marginally significant main effect of deprivation stage (*F* (1, 8) = 4.858, *p* = 0.059). Furthermore, there were significant interactions between noise level and deprivation stage (*F* (1, 8) = 5.744, *p* = 0.043) and between noise level and spatial frequency (*F* (9, 72) = 122.368, *p* < 0.001) but not between deprivation stage and spatial frequency (*F* (9, 72) = 0.983, *p* = 0.461). The interaction among noise level, deprivation stage, and spatial frequency was not significant (*F* (9,72) = 1.315, *p* = 0.244). Further least significant difference (LSD) analysis showed that in zero noise, the CS after MPD was significantly higher than that before MPD (*p* = 0.048), but in high noise, the CS was comparable before and after MPD (*p* = 0.729). Above all, these results implied that MPD boosted CS in the deprived eye at all spatial frequencies.

We conducted a Pearson correlation analysis on log spatial frequency and log CS improvement at the zero-noise level. The results showed that the two factors were significantly correlated with each other (*r* = 0.810, *p* = 0.004), indicating that high spatial frequency produces larger CS improvement. It is worth to note that the estimated threshold at 16 cpd before MPD was close to 1, which may underestimate the CS improvement. However, even after we drop this point, the correlation was still strong (*r* = 0.925, *p* < 0.001). Thus, our conclusion is reliable.

We estimated the area under the log CSF (AULCSF) (Figure 3B) to index CS over all spatial frequencies (Ramamurthy and Blaser, 2018, 2021; Yan et al., 2020; Zhang et al., 2021). A repeated-measures ANOVA on AULCSF with external noise level (zero vs. high) and deprivation stage (before vs. after) as within-subject factors showed a significant main effect of noise level (*F* (1, 8) = 43.008, *p* < 0.001) instead of deprivation stage (*F* (1, 8) = 4.852, *p* = 0.059). There was a significant interaction between noise level and deprivation stage (*F* (1, 8) = 6.548, *p* = 0.034). At the zero-noise level, the AULCSF after MPD was higher than that before MPD (7.158 ± 0.933 vs. 5.480 ± 0.338, mean ± SE, *p* = 0.044) in the LSD test. However, at the high-noise level, AULCSF before and after MPD were comparable (2.401 ± 0.289 vs. 2.349 ± 0.321, *p* = 0.711). These results further confirmed the positive effect of MPD on CS.

**Figure 4:**
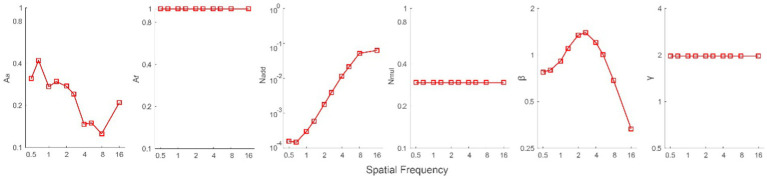
*A*_a_, *A*_f_, *N*_add_, *N*_mul_, *β*, and *γ* as a function of spatial frequencies from the best-fitting model (M1).

To investigate the mechanisms underlying the increase in CS, data from all subjects were averaged (geometric mean) prior to fitting them with the PTM. The paired-sample *t*-test showed no significant difference in the slopes of psychometric functions before and after MPD (*t* (8) = 0.154, *p* = 0.881, see details in Supplementary material). That is, the internal multiplicative noise remained constant after MPD, and we only set *A*_a_ and *A*_f_ free. There were four possible models: both *A*_a_ and *A*_f_ were changed by MPD (M0), and this model contains 42 parameters, including 10 *N*_add_, 10 *A*_a_, 10 β, 10*A*_f_, 1 *N*_mul_, and 1 γ; only *A*_a_ was changed by MPD (M1), and this model contains 32 parameters, including 10 *N*_add_, 10 *A*_a_, 10 β, 1 *N*_mul_, and 1 γ; only *A*_f_ was changed by MPD (M2), and this model contains 32 parameters, including 10 *N*_add_, 10 β, 10 *A*_f_, 1 *N*_mul_, and 1 γ; and both *A*_a_ and *A*_f_ were constant (M3), and this model contains 22 parameters, including 10 *N*_add_, 10 β, 1 *N*_mul_, and 1 γ. The *r*^2^ of these four models was 98.53, 98.53, 73.63 and 72.88%, respectively. The *r*^2^ values of M0 and M1 were significantly higher than those of M2 and M3 (all *p* < 0.05), and there was no significant difference between M0 and M1 or between M2 and M3 (all *p* > 0.05). Owing to fewer parameters in M1 than in M0, we selected M1 as the best-fitting model for further analysis. Average across all spatial frequencies, the mean of log *A*_a_ in M1, was −0.639 ± 0.053 (Figure 4). Because *A*_a_ is the coefficient of internal additive noise, our results indicated that decreased internal additive noise contributes to CS increases after MPD.

In addition, we conducted a Pearson correlation analysis on log spatial frequency and log *A*_a_. The results showed that the two factors were significantly correlated with each other (*r* = −0.787, *p* = 0.007), indicating that internal additive noise was decreased more at high spatial frequencies after MPD. We also reexamined it after dropping the data at 16 cpd, and found that the correlation remained strong (*r* = −0.922, *p* < 0.001).

## Discussion

Our study systematically investigated the effect of 150 min MPD on CSF and further determined the corresponding mechanisms with the PTM. Consistent with previous results, CS gain was observed (Zhou et al., 2013a). However, we found that the CS gain simply occurred when external noise was absent and was spatial frequency dependent. The PTM analysis revealed that the decrease in internal additive noise underlies the MPD-induced CS improvement with a larger decrease at high spatial frequencies.

To date, many studies have examined MPD-induced visual function gain, but the current experiment still offers important innovation. First, we measured the CSF changes before and after deprivation using the qCSF method, which has high accuracy, efficiency and test–retest reliability (Hou et al., 2010; Kim et al., 2018). Traditional CSF measurements over 5–10 spatial frequencies require approximately 500–1,000 trials to have reasonable precision (Harvey, 1986), which is time-consuming. Notably, the peak effect of MPD may occur in the first 10 min after patch removal (Lunghi et al., 2011, 2013; Zhou et al., 2013a,b). Thus, the qCSF procedure can capture the maximum power of MPD-induced CS improvement. In addition, the MPD effect can be evaluated at a wide range of spatial frequencies (as well as AULCSF) rather than a single spatial frequency in previous studies (Zhou et al., 2013a). A recent study involving 18 patients with amblyopia (Zhou et al., 2019) suggested that a two-hour daily monocular deprivation for up to 2 months can help patients recover their binocular visual functions and visual acuity in the amblyopic eye. However, the indexes the authors used to assess visual recovery were only for suprathreshold stimuli. Therefore, future studies may apply the qCSF method to assess the cumulative improvement in near-threshold patients caused by 2 h/day of MPD for 2 months. In our study, the CS improvement was more profound at high spatial frequencies, which is good news for clinical implementation. Since the defects of amblyopia on CSF mainly focus on high spatial frequencies, MPD treatment is helpful.

In our study, MPD failed to increase the CS when external noise was present, indicating that the MPD effect was not universal. With the help of the PTM, we explored the psychophysical mechanism of CS improvement from a modeling point of view. First, decreased internal additive noise accounted for MPD-induced CS improvement. Internal additive noise refers to the random fluctuations in neural activity within the visual system that can affect the processing of visual information. It is considered a source of noise that can degrade the quality of visual perception. MPD improves the signal-to-noise ratio of sensory responses, making it easier to detect which interval contain a grating. Neurons in the primary visual cortex are specifically tuned to respond to variations in contrast. Thus, MPD improved CS by reducing the random fluctuations in neural activity in primary visual cortex. In addition, our results may be explained by the changes in GABAergic inhibition in the visual cortex. It has been demonstrated that GABA and its agonists improve visual cortical function by increasing the signal-to-noise ratio (Leventhal et al., 2003). Lunghi et al. (2015) found that GABA concentrations measured during monocular stimulation correlated with deprived eye dominance after MPD (Lunghi et al., 2015). Second, the decrease in internal additive noise was more profound at high spatial frequencies, which suggested that the MPD effect was modulated by spatial frequency. In the future, studies to integrate the psychophysical and physiological mechanisms of MPD are necessary.

The current study utilizes external noise paradigms to characterize changes in visual performance (Xu et al., 2006; Lu and Dosher, 2008; Bower and Andersen, 2012; Zhang et al., 2018, 2022; Guo et al., 2023). It operates under the assumption of noise-invariant processing, where the processing properties remain unchanged in the presence and absence of external noise. Previous studies have reported that this assumption does not hold when the noise is spatiotemporally localized to the target, but it remains valid when the noise is spatiotemporally extended (Allard and Cavanagh, 2011; Allard et al., 2013; Allard and Faubert, 2013, 2014a,b). However, these studies employed motion discrimination, relied on a peripheral phenomenon with limited understanding, or focused on specific noise types. A recent study utilizing a standard contrast detection task in white noise demonstrated that temporally extended external noise aligns the properties with internal noise, indicating the absence of processing changes (Silvestre et al., 2017). In the current study, two key methodological choices were made. First, we presented the external noise 70 msec before the target and made it appear larger than the target, even though it did not follow the typical spatiotemporal extension. Second, we explicitly instructed the subjects to adopt a detection processing strategy instead of a recognition strategy. Based on these choices, it can be assumed that the observer did not utilize the temporal window information of the noise, thereby supporting the validity of the assumption of noise-invariant processing.

To ensure that CSF measurement was conducted during the peak period of the MPD effect, we exclusively considered the monocular task. Future studies should aim to investigate the systematic changes in binocular contrast sensitivity and assess the generalizability of these effects to other deprivation methods.

## Data availability statement

The raw data supporting the conclusions of this article will be made available by the authors, without undue reservation.

## Ethics statement

The studies involving human participants were reviewed and approved by Ethical Review Committee of Hebei Normal University. The patients/participants provided their written informed consent to participate in this study.

## Author contributions

PZ designed the experiment. JinwL and ZC collected and analyzed the data. JinwL and PZ wrote the manuscript. PZ, DW, JinwL, ZC, JingL, LL, LC, JT, and ZW revised the manuscript. All authors contributed to the article and approved the submitted version.

## Funding

This work was supported by the Natural Science Foundation of Hebei Province (C2021205005 to PZ), Science Foundation of Hebei Normal University (L2022B26 to PZ), and University-level scientific research project in CDMC (202113 to JT).

## Conflict of interest

The authors declare that the research was conducted in the absence of any commercial or financial relationships that could be construed as a potential conflict of interest.

## Publisher’s note

All claims expressed in this article are solely those of the authors and do not necessarily represent those of their affiliated organizations, or those of the publisher, the editors and the reviewers. Any product that may be evaluated in this article, or claim that may be made by its manufacturer, is not guaranteed or endorsed by the publisher.
