# Supplementary material for: Short-term monocular pattern deprivation reduces the internal additive noise of the visual system
Source: Front Neurosci. 2023 Jul 27;17:1155034. doi: 10.3389/fnins.2023.1155034 (PMC10426733; doi:10.3389/fnins.2023.1155034)
Supplement: Supplementary file 1 [file Data_Sheet_1.docx]

**Supplementary Information**

***Slope Check in Measurement of the Contrast Sensitivity Function***

Here we used the quick contrast sensitivity function (qCSF) method, which assumes that the slope of the psychometric function was invariant across spatial frequencies, external noise level, and before and after deprivation, to measure the contrast sensitivity functions (CSFs). The existing studies have proved that the slope of the psychometric function did not change across spatial frequencies and external noise levels (Chen et al., 2014; Hou et al., 2015). Thus, this study compared the slopes of the psychometric function before and after monocular pattern deprivation (MPD) to test whether they were consistent. The performance of an observer was computed by the following equation:

$P_{i,j}\left( x \right)=\gamma+\left( 1-\gamma-\lambda\right)\left( 1-exp\left( -{10}^{s\left( {log}_{10}\left( x \right)-{log}_{10}\left( \tau_{i,j} \right) \right)} \right) \right)$, Eq. 1

where *P_i,j_*(*x*) is percent correct in the *i*^th^ spatial frequency and *j*^th^ external noise level; guessing rate (*γ*) is 0.5 and lapse rate (*λ*) is 0.02; *τ* is the contrast threshold at 80.3% correct performance level; the unique free parameter is *s*, which denotes the slope of psychometric function.

The raw data from the qCSF measurements before and after MPD consisted of stimulus condition (spatial frequency, external noise condition, and stimulus contrast) and response accuracy (correct or incorrect) in each trial.

The paired-sample T test was performed on log slope with deprivation stage (before and after) as within subject variable. Averaged over observers, the slope of the best-fitting model is 0.270 ± 0.039 before MPD and 0.257±0.046 after MPD, with no significant difference (t(8) = 0.217, *p* = 0.834). Our results indicated that *A_m_* should be removed from PTM, which consistent with those in earlier studies (Chen et al., 2014; Hou et al., 2015; Yan et al., 2020; Zhang et al., 2018, 2021).

**References**

Chen, G., Hou, F., Yan, F.-F., Zhang, P., Xi, J., Zhou, Y., Lu, Z.-L., & Huang, C.-B. (2014). Noise Provides New Insights on Contrast Sensitivity Function. *PLoS ONE*, *9*(3), e90579. https://doi.org/10.1371/journal.pone.0090579

Hou, F., Lesmes, L., Bex, P., Dorr, M., & Lu, Z.-L. (2015). Using 10AFC to further improve the efficiency of the quick CSF method. *Journal of Vision*, *15*(9), 2. https://doi.org/10.1167/15.9.2

Yan, F.-F., Hou, F., Lu, H., Yang, J., Chen, L., Wu, Y., Chen, G., & Huang, C.-B. (2020). Aging affects gain and internal noise in the visual system. *Scientific Reports*, *10*(1), 6768. https://doi.org/10.1038/s41598-020-63053-0

Zhang, P., Hou, F., Yan, F.-F., Xi, J., Lin, B.-R., Zhao, J., Yang, J., Chen, G., Zhang, M.-Y., He, Q., Dosher, B. A., Lu, Z.-L., & Huang, C.-B. (2018). High reward enhances perceptual learning. *Journal of Vision*, *18*(8), 11–11. https://doi.org/10.1167/18.8.11

Zhang, P., Wang, H., Ren, W., Lu, Q., Li, C., Chen, G., Zhang, S., Tao, J., Li, Y., Wu, D., & Wang, Z. (2021). Lower Internal Additive Noise and Better Perceptual Template Characterize Binocular Contrast Sensitivity Summation. *Frontiers in Psychology*, *12*, 740759. https://doi.org/10.3389/fpsyg.2021.740759
